# Semantic segmentation guided detector for segmentation, classification, and lesion mapping of acute ischemic stroke in MRI images

**DOI:** 10.1016/j.nicl.2022.103044

**Published:** 2022-05-12

**Authors:** Yi-Chia Wei, Wen-Yi Huang, Chih-Yu Jian, Chih-Chin Heather Hsu, Chih-Chung Hsu, Ching-Po Lin, Chi-Tung Cheng, Yao-Liang Chen, Hung-Yu Wei, Kuan-Fu Chen

**Affiliations:** a Department of Neurology, Chang Gung Memorial Hospital, Keelung, Taiwan; b Institute of Neuroscience, National Yang Ming Chiao Tung University, Taipei, Taiwan; c Community Medicine Research Center, Chang Gung Memorial Hospital, Keelung, Taiwan; d College of Medicine, Chang Gung University, Taoyuan, Taiwan; e Clinical Informatics and Medical Statistics Research Center, Chung Gung University, Taoyuan, Taiwan; f Center for Geriatrics and Gerontology, Taipei Veterans General Hospital, Taipei, Taiwan; g Institute of Data Science, National Cheng Kung University, Tainan, Taiwan; h Department of Trauma and Emergency Surgery, Chang Gung Memorial Hospital, Linkou, Taiwan; i Department of Radiology, Chang Gung Memorial Hospital, Keelung, Taiwan; j Department of Medical Imaging and Radiological Sciences, Chang Gung University, Taoyuan, Taiwan; k Department of Electrical Engineering, National Taiwan University, Taipei, Taiwan; l Department of Emergency Medicine, Chang Gung Memorial Hospital, Keelung, Taiwan

**Keywords:** AIS, Acute Ischemic Stroke, MRI, Magnetic Resonance Imaging, PACS, Picture Archiving and Communication System, DWI, Diffusion-Weighted Imaging, T1W, T1-Weighted imaging, SGD-Net, Semantic Segmentation Guided Detector Network, SGD-Net Plus, Semantic Segmentation Guided Detector Network Plus, SegMap, Segmented lesion Map, T2W, T2-weighted imaging, FLAIR, FLuid-Attenuated Inversion Recovery, DICOM, Digital Imaging and Communications in Medicine, NIFTI, Neuroimaging Informatics Technology Initiative, S1, Stage-One, S2, Stage-Two, MPRAGE, Magnetization Prepared RApid Gradient Echo, MNI, Montreal Neurological Institute, VGG, Visual Geometry Group, ResNet, Residual Net, DenseNet, Dense Convolutional Network, 3D-CNNs, Three-Dimensional Convolutional Neural Networks, IoU, Intersection-over-Union, AUROC, the Area Under the Receiver Operating Characteristics, AUPRC, the Area Under the Precision-Recall Curve, ADC, Apparent Diffusion Coefficient, SGD-net, SGD-net plus, Acute ischemic stroke, Diffusion-weighted imaging, Joint segmentation and classification, Lesion distribution and mapping

## Abstract

**Background and purpose:**

MRI images timely and accurately reflect ischemic injuries to the brain tissues and, therefore, can support clinical decision-making of acute ischemic stroke (AIS). To maximize the information provided by the MRI images, we leverage deep learning models to segment, classify, and map lesion distributions of AIS.

**Methods:**

We evaluated brain MRI images of AIS patients from 2017 to 2020 at a tertiary teaching hospital and developed the Semantic Segmentation Guided Detector Network (SGD-Net), composed of the first U-shaped model for segmentation in diffusion-weighted imaging (DWI) and the second model for binary classification of lesion size (lacune vs. non-lacune) and circulatory territory of lesion location (anterior vs. posterior circulation). Next, we modified the two-stage deep learning model into SGD-Net Plus by automatically segmenting AIS lesions in DWI images and registering the lesion in T1-weighted images and the brain atlases.

**Results:**

The final enrollment (216 patients with 4606 slices) was divided into 80% for model development and 20% for testing. S1 model segmented AIS lesions in DWI images accurately with a pixel accuracy > 99% (Dice 0.806–0.828 and IoU 0.675–707). In comprehensive evaluation of classification performance, the two-stage SGD-Net outperformed the traditional one-stage models in classifying AIS lesion size (accuracy 0.867–0.956 vs. 0.511–0.867, AUROC 0.962–0.992 vs. 0.528–0.937, AUPRC 0.964–0.994 vs. 0.549–0.938) and location (accuracy 0.860–0.930 vs. 0.326–0.721, AUROC 0.936–0.988 vs. 0.493–0.833, AUPRC 0.883–0.978 vs. 0.365–0.695). The precise lesion segmentation at the first stage of the deep learning model was the basis for further application. After that, the modified two-stage model SGD-Net Plus accurately reported the volume, region percentage, and lesion percentage of each region on the selected brain atlas. Its reports provided clear descriptions and quantifications of the AIS-related brain injuries on white matter tracts, Brodmann areas, and cytoarchitectonic areas.

**Conclusion:**

Domain knowledge-oriented design of artificial intelligence applications can deepen our understanding of patients’ conditions and strengthen the use of MRI for patient care. SGD-Net precisely segments AIS lesions on DWI and accurately classifies the lesions. In addition, SGD-Net Plus maps the AIS lesions and quantifies their occupancy in each brain region. They are practical tools to meet the clinical needs and enrich educational resources of neuroimage.

## Highlights

• MRI images provide a wealth of information about acute ischemic stroke (AIS).
• SGD-Net uses a two-stage design to segment and classify AIS lesions in MRI images.
• SGD-Net as a lesion-based classifier outperforms traditional one-stage models.
• SGD-Net Plus applies multimodal images to quantify AIS lesion distribution.
• SGD-Net and SGD-Net Plus automatically segment, classify, and maps AIS lesions.

## Graphical abstract

**Figure:**
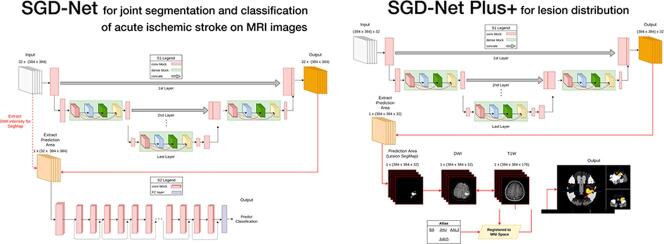

## 1 Introduction

Stroke is a leading cause of healthcare burden globally, and around 70% of stroke cases are ischemic strokes. Every year, 6 million new ischemic stroke cases lead to 6 million disability-adjusted life years (Feigin et al., 2017). The estimated lifetime risk of ischemic stroke after 25 years of age is 18.3%, with significant regional differences and the highest risk in East Asia (38.8%) (Collaborators et al., 2018). Contemporary medical doctors face challenges in timely and accurate determination of acute stroke lesions, predicting changes in acute neurological symptoms, and providing individualized precision medical treatment to patients with acute ischemic stroke (AIS).

Magnetic resonance imaging (MRI) detects AIS and supports the clinical decision-making process in AIS by applying multiple sequences to collect comprehensive information simultaneously. It helps clinicians precisely locate AIS lesions (Davis et al., 2006), grade severity (Kellner et al., 2019), and predict tissue survival (Yu et al., 2020) and clinical outcomes (Schaefer et al., 2015). However, for non-radiologist clinical professionals, the knowledge required for proper interpretation of MRI images limits the optimal utilization of instantaneously available images on the Picture Archiving and Communication System (PACS).

Segmentation is the central task of machine learning in AIS, especially when using deep learning to recognize lesions (Akkus et al., 2017). Previous studies have tried to use multimodal MRI images to segment AIS lesions (Winzeck et al., 2018). However, a single valid MRI sequence can be sufficient to show AIS lesions. Therefore, a growing number of studies have used diffusion-weighted MRI to reveal AIS lesions because the corresponding changes of the water-restricted AIS lesions appear timely, sensitively, and precisely in diffusion-weighted imaging (DWI) images (Davis et al., 2006). However, derivative clinical applications of DWI-based AIS lesion segmentation are still under development. Only a few research groups have applied deep learning-assisted segmentation in scoring ischemic damage on brain tissue (Yoshimoto et al., 2019) or performing stroke phenotyping (Wu et al., 2019) based on DWI images.

This study leveraged deep learning models to transform MRI images into real-time knowledge to realize explainable artificial intelligence (AI). The directly connected two-stage Semantic Segmentation Guided Detector Network (SGD-Net) utilized 2D segmentation and 3D classification on AIS DWI to determine lesion size and location. Furthermore, the modified two-stage Semantic Segmentation Guided Detector Network Plus (SGD-Net Plus) model registered the segmented AIS lesions to T1-weighted imaging (T1W) images and the standard space to calculate their distribution on anatomical regions to provide clinical physicians with helpful information.

## 2 Methods

### 2.1 Database

DWI images of adult patients with a clinical diagnosis of AIS confirmed by neurologists were obtained from 2017 to 2020. Only patients with MRI acquisition<14 days after stroke onset were included in the study. All MRI images were acquired using a Siemens Magneton Skyra 3 T scanner (Siemens Healthineers, Germany) at Keelung Chang Gung Memorial Hospital, a tertiary teaching hospital in Taiwan. The data were anonymized and de-identified before entering the raw digital medical imaging database. Our institutional review board approved this study (approval numbers 202101915B0).

### 2.2 AIS lesion labeling

The image review board was composited of three members and directed by neurologists. The ITK-SNAP toolbox (https://www.itksnap.org) was used to manually label AIS lesions on DWI to create a segmented lesion map (SegMap). Each image was reviewed by three members of the image review board to reach an agreement by consensus. The three board members agreed on AIS lesion segmentation and classifications after a comprehensive review of structural MRI, including T1W, T2-weighted imaging (T2W), fluid-attenuated inversion recovery (FLAIR) images, formal imaging reports of radiologists, and the diagnoses at hospital discharge. In addition, during image review and labeling, patients with other structural lesions (e.g., brain tumor or post-surgical encephalomalacia), other acute brain diseases (e.g., hemorrhagic stroke or meningitis), or motion artifacts were excluded. In addition, we excluded images without any recognizable lesions for the AIS.

The first target of classification was the size of AIS lesions in binary classes: lacune and non-lacune. The definition of lacune was an isolated small (<20 mm) water restricted lesion in DWI images, and the lesion was explainable to the patient’s AIS symptoms (Arsava et al., 2010, Wardlaw, 2005). Any other multiple scattered small lesions were not considered lacunes because they were highly possible from embolic origin but not small vessel occlusion (Wessels et al., 2005). The second classifying target was the vascular territory of the AIS lesion by anterior and posterior circulatory territories. The references of classification were based on the atlas by Tatu et al. and its adaptation to multimodal MRI (Kabir et al., 2007, Tatu et al., 1998). In image labeling, we made extra annotations for the patients with lesions in both the anterior and posterior circulatory. Those patients were excluded from classifying lesion location because the binary classification did not allow an answer true for both classes.

### 2.3 Image preprocessing

DWI images were exported from Digital Imaging and Communications in Medicine (DICOM) to Neuroimaging Informatics Technology Initiative (NIFTI) format. The image scale of DWI was 384 × 384 pixels per transverse slice and from 20 to 28 serial transverse slices per patient (spatial resolution = 0.57 × 0.57 × 7 mm; b value = 1000 s/mm^2^). The image set for each patient was replenished with blank images to 32 slices to maintain the consistency of the image input size. The DWI intensity of the raw images was re-scaled by z-score normalization. In addition, data augmentation was performed on Albumentations (https://albumentations.ai) with functions of rotation, horizontal flip, and shift-scale-rotate (Buslaev et al., 2020).

### 2.4 SGD-net

#### 2.4.1 Two-stage SGD-net model development

**Fig. 1:**
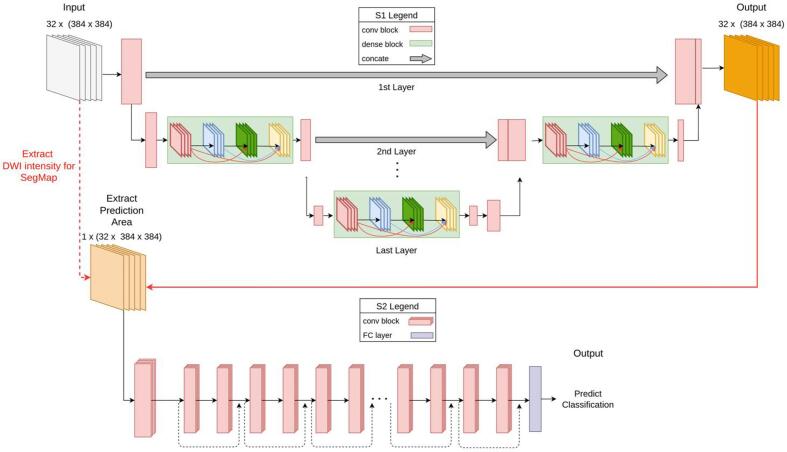
The architecture of SGD-Net. The S1 architecture of the SGD-Net is a modification of U-Net from VGG, DenseNet, and ResNet to form VGGUNet, Dense-UNet, and Res-UNet. The DWI inputs passed through a hierarchical contraction path and a corresponding extraction path. The illustration describes the combination of DenseUNet121 in S1 and 3D-ResNet18 in S2. Along the paths, the convolution blocks are connected to the pooling of dense blocks. Every convolutional block contains convolution layers, a rectified linear unit (ReLu), and a batch normalizer as the basic unit. The hierarchical concatenation between contraction and extraction pathways is pooled into the output layer, and the images are exported in the same size as the input images. Finally, the extracted stroke area images are input to the fully connected (FC) convolutional neural networks S2 to classify AIS lesion size and location.

SGD-Net contained a U-shaped Stage-One (S1) for segmentation and a fully connected Stage-Two (S2) for classification (Fig. 1). U-Net was the central architecture for constructing the S1 model (Ronneberger et al., 2015). We tested several backbones for S1 modeling, including the convolutional network of the Visual Geometry Group (VGG) (Simonyan and Zisserman, 2015), Residual Net (ResNet) (He et al., 2016), and Dense Convolutional Network (DenseNet) (Huang et al., 2017). They were modified into U-shaped DenseUNet (Li et al., 2018), ResUNet (Diakogiannis et al., 2020), and VGGUNet (Ghosh et al., 2021). The segmentation in S1 was based on 2D images of each DWI slice. The model later composed all the slices of each patient and yielded a prediction of SegMap.

The S2 classified AIS lesions based on the predicted SegMap from S1. Image sets per patient defined the classification to consider the three-dimensional construction of AIS lesions. The S2 backbones of comparisons were 3D-ResNet18, 3D-ResNet50 (He et al., 2016), and three-dimensional convolutional neural networks (3D-CNNs) (Zunair et al., 2020) (Table 1).

**Table 1:** Backbones of the machine learning models.

		Model	Data type	Backbone	Epochs	Loss function
Two-stage model (SGD-Net)	S1	VGGUNet	2D MRI	VGG16	150	Focal Tversky Loss
		ResUNet	2D MRI	ResNet50	150	Focal Tversky Loss
		DenseUNet	2D MRI	DenseNet121	150	Focal Tversky Loss
	S2	3D-CNNs	3D MRI	CNNs	100	Focal Loss
		3D-ResNet50	3D MRI	ResNet50	100	Focal Loss
		3D-ResNet18	3D MRI	ResNet18	100	Focal Loss
One-stage model		3D-ResNet50	3D MRI	ResNet50	100	Focal Loss
		3D-ResNet18	3D MRI	ResNet18	100	Focal Loss
		3D-CNNs	3D MRI	CNNs	100	Focal Loss

S1, stage 1 for segmentation; S2, stage 2 for classification. The loss function of the one-stage and two-stage models was based on focal loss for imbalanced classes of data. In addition, for S1 of the two-stage models, the Tversky index was combined with focal loss to form focal Tversky loss for enhanced learning for lesion segmentation in the U-Net structure.

#### 2.4.2 One-stage model

One-stage 3D models were the traditional design for classifying 3D objects. We tested the performance of 3D-ResNet18, 3D-ResNet50 (Hara et al., 2017), and 3D-CNNs (Zunair et al., 2020) to compare their performance with the two-stage SGD-Net model (Table 1).

#### 2.4.3 Model tuning

Focal Tversky Loss (Abraham and Khan, 2019) in S1 and Focal Loss in S2 (Lin et al., 2020) further tuned model hyperparameters. Focal Tversky Loss contained Tversky Index (TI) for weighting the ratio of true positives (TPs) among the linear combination of TP, false-negative (FN), and false-positive (FP):

$$\mathrm{TverskyIndex}=TI=\frac{\mathrm{TP}}{\mathrm{TP}+aFN+\beta FP}$$

$$FocalTverskyLoss\left(p_{t}\right)={\left(1-TI\right)}^{r}$$

Focal Loss function contained a modulating factor (1 − *p_t_*)*^γ^* to the cross-entropy loss, a focusing parameter *γ*, and a balancing variant *α* and was a guide for fine-tuning the hyperparameter:

$$FocalLoss\left(p_{t}\right)=-a{\left(1-p_{t}\right)}^{r}log\left(p_{t}\right)$$

Adam optimizer with an adaptive learning rate by cosine annealing optimized model performance during training (Kingma and Ba, 2015). In addition, the highest score of Youden's Index of each model determined its threshold (Ruopp et al., 2008).

#### 2.4.4 Model performance evaluation

Performance analyses were on different bases for the two stages: S1 performance was based on pixels, and S2 performance was based on patients. So first, we used the confusion matrix to evaluate S1 semantic segmentation including pixel accuracy, intersection-over-union (IoU), and Dice coefficient. Pixel accuracy was the percentage of the area of correct segmentation prediction over the ground truth, i.e., the pixel sum of TP and TN divided by the pixel sum of TP, FP, true negative (TN), and FN:

$$\mathrm{Pixelaccuracy}=\frac{\mathrm{areaofcorrectprediction}}{\mathrm{totalareaofprediction}}=\frac{\mathrm{TP}+TN}{\mathrm{TP}+FP+TN+FN}$$

IoU and Dice were defined as follows:

$$\mathrm{IoU}=\frac{\mathrm{areaofoverlap}}{\mathrm{areaofunion}}$$

$$\mathrm{Dice}=\frac{2\times areaofoverlap}{\mathrm{totalareofpredictedsegmentationandgroundtruth}}$$

Next, the performance comparison of S2 classifiers was evaluated by the area under the receiver operating characteristics (AUROC) and the area under the precision-recall curve (AUPRC) (Saito et al., 2015).

#### 2.4.5 Data for development and testing of the SGD-Net

The dataset was chronologically separated into 80% images acquired from 2017 to early 2020 for model development and 20% images in late 2020 for testing. The test dataset contained separate images from different patients and did not overlap the development dataset for validation. In addition, the development dataset was further randomly divided into 80% for training and 20% for internal validation.

#### 2.4.6 SGD-Net plus

**Fig. 2:**
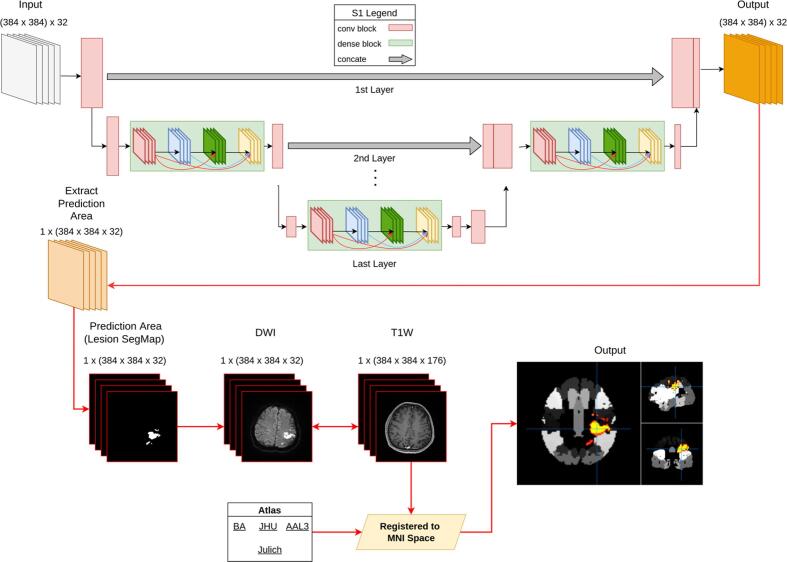
Design of SGD-Net Plus. SGD-Net Plus connected the S1 of SGD-Net and serial multimodal imaging processes. The AIS lesion SegMap was projected back to the DWI images and co-registered with T1W images and brain atlases on standard space of the Montreal Neurological Institute (MNI). The model calculated the percentage of each brain region affected by the AIS lesions and the percentage of AIS lesion volume in each brain region. The brain atlases used in SGD-Net Plus were Brodmann Area (BA) for functional cortical segmentation, Johns Hopkins University (JHU) white matter atlas for white matter tract distribution, Anatomical Automated Anatomical Labeling 3 (AAL3), and Julich Brain Cytoarchitectonic Atlas for anatomical brain segmentation.

SGD-Net Plus was modified from SGD-Net by preserving the two-stage design and combining multimodal imaging to identify the anatomical spaces occupied by the AIS lesions (Fig. 2). The model first generated an AIS lesion SegMap from DWI by the S1 model and then registered the SegMap to standard space on T1W images and brain atlases. Because the S1 model had already completed segmentation from the DWI image and the standard space of the brain served as a platform for co-registration, the model allowed using multi-modal images with different dimensions and resolutions. After co-registration, the lesion mapping used known brain atlases as ground truth.

The image registration process was applied as follows: (1) skull removal was applied on all T1W images. We used T1 magnetization prepared rapid gradient echo (MPRAGE) images in this study. The dimension of MPRAGE images was 256x256x176, the spacing was 0.9x0.9x1mm. (2) T1W images were co-registered to the corresponding DWIs using linear registration. (3) brain extracted T1W images were nonlinear registered and spatially normalized to the Montreal Neurological Institute (MNI) space. (4) by multiplying two transformation matrices from step 2 and 3, the atlases of Brodmann area (Strotzer, 2009), Johns Hopkins University white matter atlas (Mori et al., 2008), Automated Anatomical Labeling 3 (Rolls et al., 2020), and Julich Brain Cytoarchitectonic Atlas (Amunts et al., 2020) were backward transformed from standard MNI space to each individual's diffusion native space. (5) three lesion-related indices were generated: (a) how many lesion voxels were located in each brain region, (b) the number of voxels was converted into the percentage of lesion distribution in each brain region, and (c) the percentage of lesion voxels in each brain region. All of the above steps were achieved using FSL (Functional Magnetic Resonance Imaging of the Brain Software Library; https://www.fmrib.ox.uk/fsl).

### 2.5 Data availability

The codes of SGD-Net and samples of AIS lesion segmentation and classification on DWI are available online (https://github.com/IlikeBB/SGD-Net). The codes and demonstrations of SGD-Net Plus are available on (https://github.com/IlikeBB/SGD-Plus).

## 3 Result

### 3.1 Dataset

**Fig. 3:**
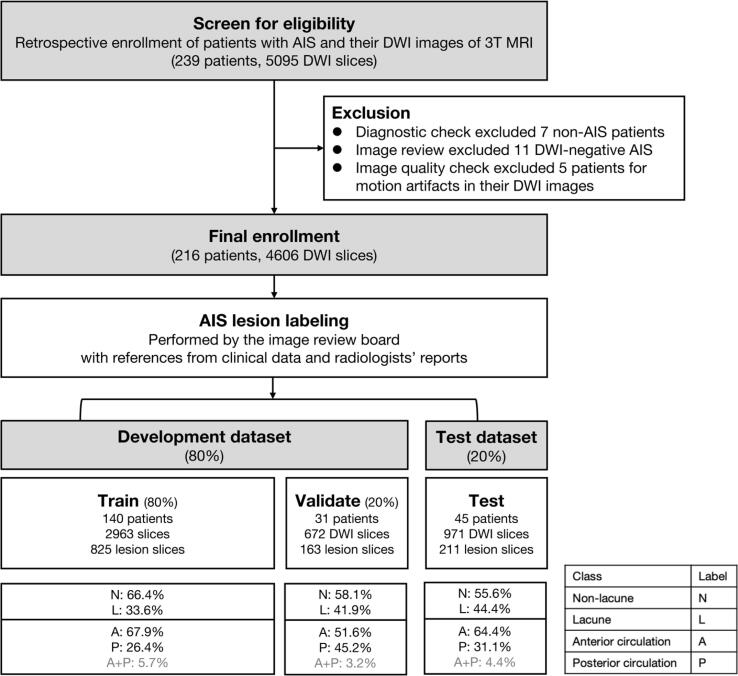
Data enrollment. Datasets were created by retrospective enrollment of patients diagnosed with AIS from 2017 to 2020. The AIS patients with brain MRI images for AIS were screened for eligibility. The final enrollment was divided chronologically into the development dataset (80%) and the test dataset (20%). The development dataset was further separated for training (80%) and validation (20%). For the classifier of the vascular territory, the image sets containing lesions in both anterior and posterior circulatory territories were excluded (5.7%, 3.2%, and 4.4% in training, validation, and testing data).

A total of 239 patients with 5095 DWI slices were enrolled for image review. Diagnostic checks excluded seven patients with non-AIS lesions. In addition, we excluded 11 patients with DWI-negative AIS (4.6%). Image quality checks further excluded five patients with images containing motion artifacts. All image labeling was agreed upon at the consensus meeting of the image review board. The final included image sets of 216 patients with 4606 slices were divided into two parts: the development dataset (80%) and the test datasets (20%) (Fig. 3).

The demographics, including age (66.32 ± 12.20 and 68.29 ± 12.58 years old, *p* = 0.338) and sex (male ratio 69.0% and 71.1%, *p* = 0.785), and the stroke-to-scan interval (4.46 ± 3.78 vs. 3.94 ± 3.61 days, *p* = 0.434) did not differ between the development and test datasets. A summary of the targets of interest for AIS lesion size revealed that the lacune and the non-lacune ratio was 35.1% to 55.6% (60 and 111 patients) in the development dataset and 55.3% to 46.7% (24 and 21 patients) in the test dataset. For the classes of lesion location, the anterior to posterior circulatory AIS ratio was 68.5% to 31.5% (111 and 51 patients) in the development dataset and 67.4% to 32.6% (29 and 14 patients) in the test dataset (Table 2).

**Table 2:** Basic information of development and test data set.

Variables		Development dataset (Train and validation)	Test dataset
Patient number		171	45
Age, mean ± std		66.32 ± 12.20	68.29 ± 12.58
Sex, male		118 (69.0%)	32 (71.1%)
Year of stroke		2017-2020	2020
Lesion size	Lacune (≤ 20 mm)	60 (35.1%)	24 (55.3%)
	Non-lacune (> 20 mm)	111 (55.6%)	21 (46.7%)
Lesion location	Anterior circulatory territory	111 (68.5%)	29 (67.4%)
	Posterior circulatory territory	51 (31.5%)	14 (32.6%)

The development dataset was composed of the training and the validation datasets. Data in patient number (%). * *p* < 0.05. ^†^ Images containing lesions in both anterior and posterior circulatory were excluded from the classification. Abbreviations: std, standard deviation.

### 3.2 Performance of SGD-Net S1 segmentation

**Fig. 4:**
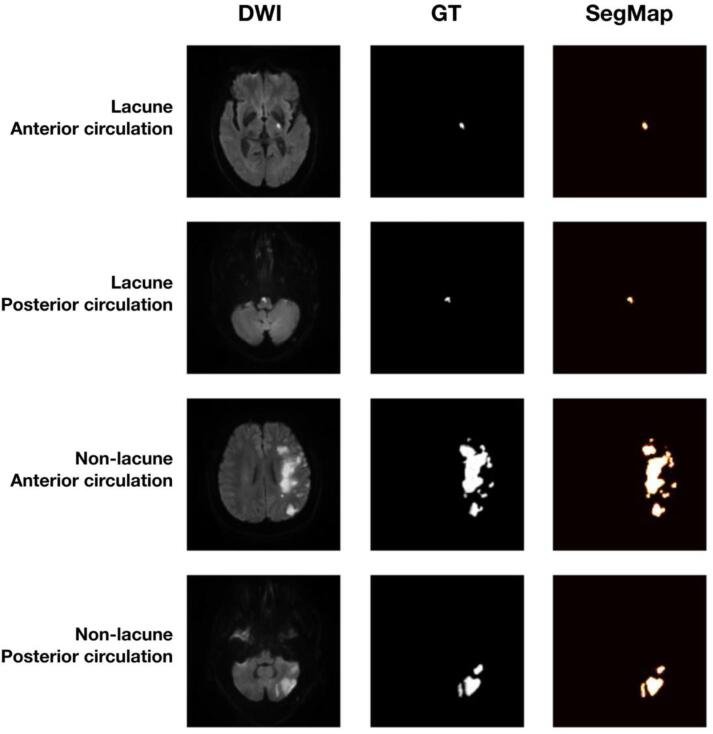
Demonstrations of SGD-Net S1 for segmentation of AIS lesions in DWI images. The four samples demonstrated the four classes of different combinations of lesion size and location. The demonstration was yielded by the SGD-Net S1 model with DenseUNet121 backbone. Ground truth was labeled by the image review board. The SegMap was the prediction by the SGD-Net S1 model. Abbreviations: GT, ground truth. SegMap: predicted segmentation map of AIS lesion.

In S1 segmentation, three backbones performed comparably to segment AIS lesion accurately, with Dice coefficient 0.813 for DenseUNet121, 0.828 for ResUNet50, and 0.806 for VGGUNet16 (Table 3**-A**). Examples of the prediction of SegMap were shown in Fig. 4.

**Table 3:** Performance of SGD-Net S1 segmentation.

A. Comparison of different backbones in SGD-Net S1 segmentation			
	Dice	IoU	Accuracy
DenseUNet121	0.813	0.685	0.999
ResUNet50	0.828	0.707	0.999
VGGUNet16	0.806	0.675	0.999

B. Test for effects of lesion size on S1 segmentation (ResUNet50)			
Small (<769 pixels)	0.761	0.614	0.999
Large (>769 pixels)	0.830	0.710	0.999

C. Test for effects of mean DWI intensity on S1 segmentation (ResUNet50)			
Low (<30.1)	0.805	0.674	0.999
High (>30.1)	0.855	0.747	0.999

D. Test for effects of training sample size (N) on S1 segmentation (ResUNet50)			
N = 20	0.668	0.502	0.999
N = 50	0.775	0.633	0.999
N = 100	0.783	0.643	0.999
N = 140 (final enrollment)	0.828	0.707	0.999

N, sample size of train dataset. IoU, intersection-over-union.

In addition, we examined if lesion size or raw DWI intensity affects model performance, using ResUNet50 as an example. The test data set was divided into two groups by the median of sum pixel or the median of mean DWI intensity of each patient. The median lesion size was 769 pixels (about 1.75 ml) in a resolution of 0.57x0.57 mm per pixel, 384x384 pixels per slice, and 7 mm thickness of a slice. The performance differences of ResUNet50 in patients with smaller (<769 pixels) and larger lesions (>769 pixels) were minor (Dice 0.761 vs. 0.830; IoU 0.614 vs. 0.710; accuracy 0.999 vs. 0.999) (Table 3**-B**). The median of each patient's average DWI intensity in the test dataset was 30.1. The performance in lower (<30.1) and higher (>30.1) DWI intensities was also similar (Dice 0.805 vs. 0.855; IoU 0.674 vs. 0.747; accuracy 0.999 vs. 0.999) (Table 3-C). Besides, we already use z-score transformation on the raw DWI intensity in image preprocessing to reduce interpersonal (inter-scan) variance. Therefore, SGD-Net S1 worked steadily without considerable size and intensity effects.

We also tested if training sample size affected performance of S1 segmentation. The performance of ResUNet50 improved steadily with training sample sizes 20, 50, 100, to 140 (Dice: from 0.668 to 0.828; IoU: from 0.502 to 0.707, accuracy: from 0.999 to 0.999) (Table 3-D). The segmentation performance seemed to be improving with currently available samples and has not yet reached a plateau. However, the accuracy was believed to be enough for us the continue the S2 classification task.

### 3.3 Performance of SGD-Net classifier

**Fig. 5:**
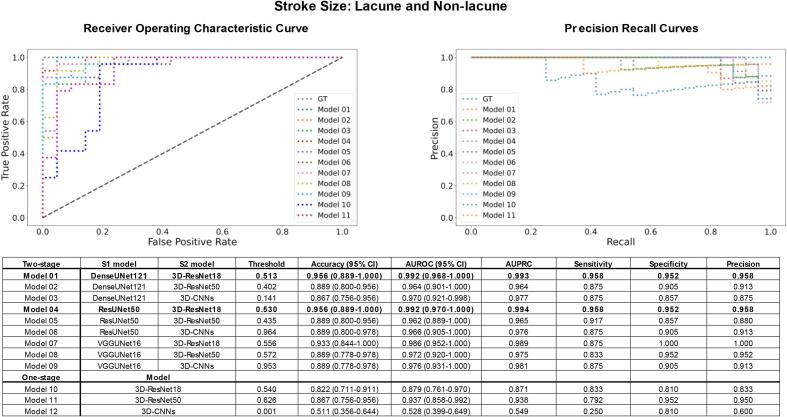
Model performance for classification of lacune and non-lacune stroke. Model performance was evaluated by accuracy, AUROC, and AUPRC. Two-stage models generally outperformed traditional one-stage models for classifying AIS lesion size as lacune and non-lacune (diameter ≤ and > 20 mm). The bold labeled the best-performed models. Model 1 (S1 DenseUNet121 + S2 3D-ResNet18) and Model 4 (S1 ResUNet50 + S2 3D-ResNet18) were the best performer. In contrast, Model 12 (one-stage 3D-CNNs) fell behind other models in classifying lesion size, and its curve was not shown in the subgraph.

Next, a comprehensive evaluation compared the performance of 11 classifiers, including nine different combinations of S1 and S2 backbones of SGD-Net and three traditional one-stage classifiers. For classification of lesion size, all the two-stage models (Model 1–9) performed well with an accuracy > 0.86, AUROC > 0.96, and AUPRC > 0.96. To specify the best model, Model 1 (composition: S1 DenseUNet121 + S2 3D-ResNet18; performance: accuracy 0.956, AUROC 0.992, AUPRC 0.993) and Model 4 (composition: S1 ResUNet50 + S2 3D-ResNet18; performance: accuracy 0.956, AUROC 0.992, AUPRC 0.994) outperformed the others. In contrast, the one-stage classifiers (Models 10–11 and 3D-CNNs) were less outstanding with an AUROC 0.528–0.937 (Fig. 5).

**Fig. 6:**
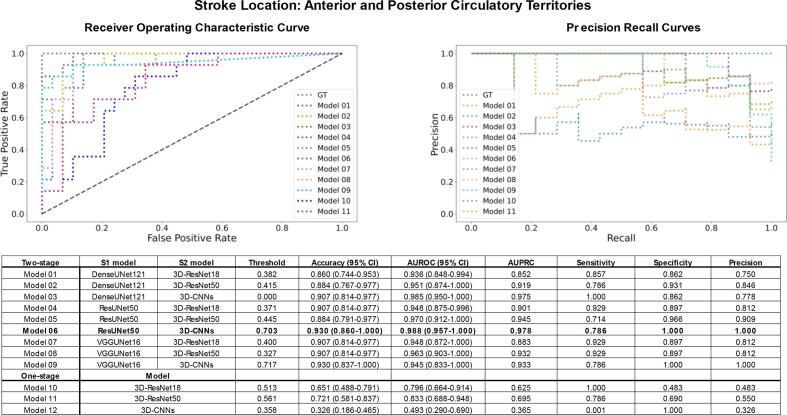
Model performance for classification of the anterior and posterior circulation. The two-stage models outperformed the one-stage models in classifying AIS lesion location in anterior or posterior circulatory territories. In addition, Model 6 (S1 ResUNet50 + S2 3D-CNNs) outperformed other models, as marked in bold. Model 12 (one-stage 3D-CNNs) performed inferior to other models in classifying circulatory territory (curve not shown in the subgraph).

In classifying the circulatory territory of AIS lesions, the two-stage models (Model 1–9) again outperformed the one-stage models (Model 10–11 and 3D-CNNs) (AUROC 0.936–0.988 vs. 0.493–0.833). For a detailed comparison of the nine SGD-Net models, Model 3 (composition: S1 DenseUNet121 + S2 3D-CNNs; performance: accuracy 0.907, AUROC 0.985, AUPRC 0.975) and Model 6 (composition: S1 ResUNet50 + S2 3D-CNNs; performance: accuracy 0.930, AUROC 0.988, AUPRC 0.978) were better than the others (Fig. 6).

### 3.4 SGD-Net plus for AIS lesion mapping

**Fig. 7:**
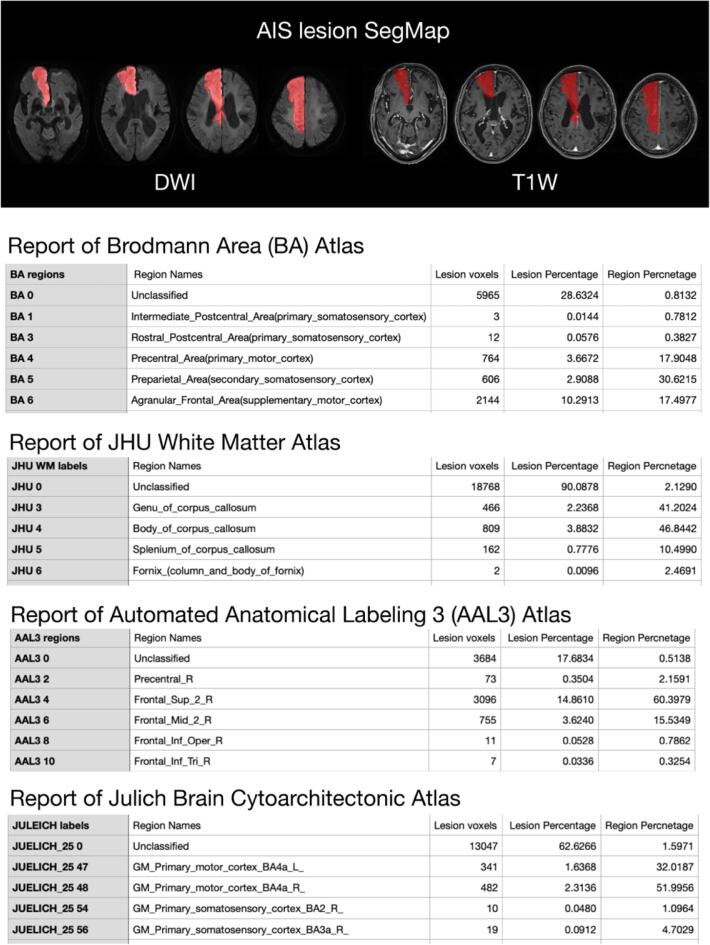
Reports of SGD-Net Plus for AIS lesion distribution. The example of the SGD-Net Plus report was a patient with acute right anterior cerebral artery territory infarction. The predicated SegMap (red) overlapped the AIS lesion on DWI images and registered on T1 MPRAGE images. The SGD-Net Plus yielded the distributions of AIS lesions on each brain atlas. The figure only showed a few lines of the report of each atlas. (For interpretation of the references to colour in this figure legend, the reader is referred to the web version of this article.)

The multimodal image analysis identified the distribution of AIS lesions on brain atlases. After that, the SGD-Net Plus yielded reports of lesion volume, lesion percentage on each region, and brain region percentages occupied by the lesion (Fig. 7).

### 3.5 User interface of SGD-net and SGD-net plus

**Fig. 8:**
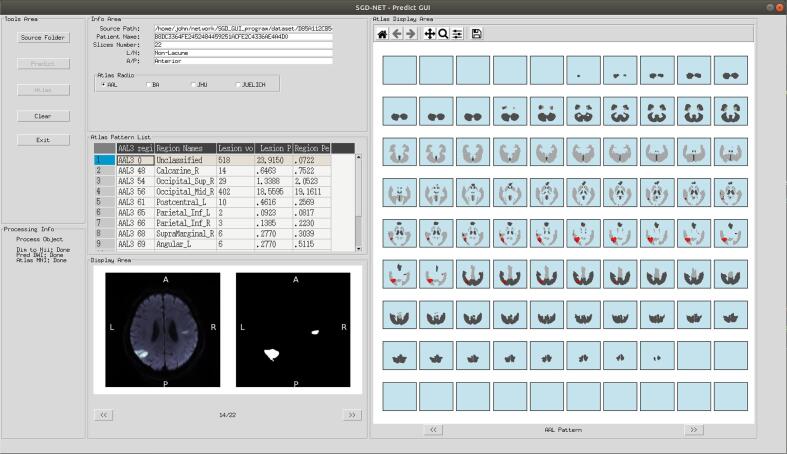
SGD-Net and SGD-Net Plus for AIS lesion report. The user interface provided the results of SGD-Net classification for AIS lesion size (lacune and non-lacune) and lesion location (anterior and posterior circulatory territory) and the results of SGD-Net Plus for AIS lesion volume in each brain region, lesion percentage in that brain region, and region percentage occupied by the lesion. It also visualized the original DWI images, S1 segmentation, and serial cuts for the atlas with red-marked AIS lesions. (For interpretation of the references to colour in this figure legend, the reader is referred to the web version of this article.)

The user interface (Fig. 8) combines the results of SGD-Net classification for AIS lesion size (lacune and non-lacune) and lesion location (anterior and posterior circulatory territory) and the reports of SGD-Net Plus for AIS lesion distribution. It also visualizes the original DWI images, the predicted AIS lesion SegMap from S1 model, and the red-marked AIS lesions on serial cuts of the brain atlases.

## 4 Discussion

### 4.1 Summary

To improve the MRI utilization in AIS by trainees, educators, students, and medical doctors who are not familiar with neuroimaging, we developed a two-stage deep learning model, SGD-Net, for the joint lesion segmentation and classification in DWI images. The two-stage models outperform the one-stage models in classifying AIS lesion size and location. The accurate segmentation in the U-shaped S1 network is the foundation of satisfactory overall performance. In addition, lesion-based classification preserves geometric and stereoscopic features of AIS lesions but avoids unnecessary learning on non-lesioned areas. It is more effective in classifying AIS than searching for important features in whole sets of images. Next, we created SGD-Net Plus by modifying the second stage of SGD-Net and applying multimodal imaging to calculate the distributions of AIS lesions on standard anatomical spaces. It provides clinical physicians valuable information to explain the patients' neurological deficits and cognitive damages.

### 4.2 DWI images in AIS study

In AIS, cytotoxic edema caused by cellular ischemia is quickly reflected in the increase in DWI signals and a decrease in apparent diffusion coefficient (ADC) signals (Albers, 1998). DWI and ADC detect AIS lesions earlier than T2W or FLAIR images (Lovblad et al., 1998). Furthermore, DWI is superior to ADC in reflecting the final lesion of AIS (Madai et al., 2014) and more sensitive and accurate than ADC for lesion segmentation in machine learning (Winzeck et al., 2019). Therefore, DWI is a promising image modality for developing image-based applications in AIS.

Previous machine learning studies developed auto-segmentation of AIS lesions on DWI images using CNNs-based models. Chen et al. used the ensembled model to segment and re-evaluate the extracted lesion area to reduce the false-positive rate and reach an acceptable performance (Dice 0.67). However, lesion size significantly affected model performance with Dice 0.61 for small and 0.83 for large lesions (Chen et al., 2017). Later, Woo et al. published the CNNs-based models with backbones of U-Net and DenseUNet. It proved that DWI-alone performed better than conventional DWI-ADC methods for AIS lesion segmentation (Dice 0.85 vs. 0.58) (Woo et al., 2019). In addition, Winzeck et al. compared DWI, ADC, DWI + ADC, and DWI + ADC + low-b diffusion images in CNNs for AIS lesion segmentation. In that study, DWI-only (Dice 0.723) outperformed ADC-only (Dice 0.564) and performed comparably with other combinations (Dice 0.756–0.789) (Winzeck et al., 2019). In our study, the SGD-Net S1 model with different backboned segmented AIS lesions used DWI-only and performed equally or better than previous studies (Dice 0.806–0.828 vs. 0.67–0.86) (Chen et al., 2017, Winzeck et al., 2019, Woo et al., 2019). Furthermore, unlike previous studies that found significant impacts on model performance by small lesion size and low DWI intensity (Chen et al., 2017, Winzeck et al., 2019), SGD-Net S1 segmentation performed steadily in conditions with either small/large lesions and low/high DWI intensities. The strategies to keep good image quality might be the reasons for this success. They were (1) enrolling acute stage images and not considering chronic stage images to prevent fading of water-restricted lesions on DWI and (2) using 3 T but not 1.5 T MRI images to promise a good signal–noise ratio. Therefore, excellent performance in segmentation is the basis for subsequent classification and lesion mapping.

### 4.3 SGD-net improved stroke classification

The novelty of this study is the merging of segmentation and classification into one model. The merging avoids learning clinically meaningless regions and has also been proven to improve classifiers' performance extensively in previous studies. For example, the segmentation-based deep fusion network (SDFN) first identified lung regions and then classified thoracic diseases based on segmentation knowledge; its AUROC of 0.815 was better than learning on an entire chest radiograph (AUROC 0.804) (Liu et al., 2019). Another case was the hierarchical classification based on the segmented brain hemorrhage lesions in computer tomography images, which reached an excellent accuracy of 94% (Shahangian and Pourghassem, 2016). Therefore, lesion-based classification is a promising method to replace traditional pixel-based classification in medical imaging analysis.

During the model development of SGD-Net, we found that the precision of segmentation in the first stage is crucial for correct classification in the successive stage. U-Net is a well-known fully convolutional network developed in 2015 to segment the limited annotated biomedical image efficiently (Ronneberger et al., 2015). The U-shaped architecture contained one symmetric contracting path and another expanding path to ensure precision encoding and decoding to preserve essential features in medical images. Because the composition of medical images is relatively concrete and straightforward and most of the features in medical images are essential, the U-shaped architecture is suitable for preserving basic and semantic features without losing necessary information. In addition, complex models in limited data can easily lead to overfitting problems. In this situation, using a simple architected U-Net can avoid the overfitting problem and achieve adequate performance.

However, precise segmentation of single slices of brain images was not sufficient to classify AIS lesions accurately. Our design of two-stage models simulated clinicians' image interpretation sequences that first focus on identifying lesions, recognizing the topologic and radiological features of the lesioned area, and then considering its spatial correlations to the whole brain (Adam et al., 2020). Thus, the S2 of SGD-Net emphasized the 3D stereoscopic features of AIS lesions by bundling DWI images of each patient. Mastery of these principles, even models with a basic structure, such as 3D-ResNet18, performed non-inferiorly to other deep layered models.

We also observed that the best S1/S2 combination differed for lesion size and vascular territory. 3D-ResNet18 is the top choice for classifying lacunes and non-lacunes, whereas 3D-CNNs are superior for anterior and posterior circulation classifications. We postulate that determining the size of targets does not mandate large, complicated networks such as 3D-ResNet50 and 3D-CNNs but only needs to stand on precisely segmented masks and classify them with simple, effective networks (such as 3D-ResNet18) to extract undistorted information (He et al., 2016). For detecting lesion location, the architecture of 3D-CNNs is advantageous for capturing spatial information, akin to the sequential graphic information of serial images. The 3D-CNNs backbone we used in this study was designed to detect tuberculosis in chest CT (Zunair et al., 2020); their simulation environment was similar to our setting, to detect solid lesions with either a single condensed, a group of satellite-like lesions, or multiple sparsely distributed nature. Therefore, goal-oriented programming based on our domain knowledge improves AI performances, and that is why 3D-CNNs-composited SGD-Net outperformed in the stereoscopic classification of AIS lesions.

### 4.4 Clinical utilities of SGD-Net plus

The initiation of developing SGD-Net Plus was based on neurologists' clinical needs for knowing the exact damages to functional or anatomical structures of the brain. In addition, multiple factors may cloud the presentations of AIS patients. For example, medical complications like infection process, abnormal blood sugar level, imbalanced electrolyte state, limb pain, or old age can increase bedside examination difficulties and mask the patients' neurological deficits (Kumar et al., 2010). In these situations, the report of SGD-Net Plus hints to the clinical physicians about potential neurologic sequelae and cognitive impairment after the acute stage of stroke. More than that, some post-stroke cognitive impairments are the distant effects of white matter tract damage and can be localized by the SGD-Net Plus (Wang et al., 2016). Physicians can be more alert to the development of post-stroke psychiatric symptoms (e.g., post-stroke depression) by knowing the damages to related brain areas and circuits (e.g., the left limbic-cortical-striatal-pallidal-thalamic circuit and its connected left amygdala and cingulum) (Terroni et al., 2011). From previous experiences, we expect future deployment of SGD-Net Plus to provide precise localization of AIS. In addition, the deep learning-assisted image-based AIS lesions analysis will help advanced evaluate stroke lesion damages and offer references to personalized post-stroke rehabilitation.

Besides, SGD-Net Plus can be a valuable medium for neuroimage education. When evaluating a stroke patient, the trainees will benefit from referencing the patient's clinical presentations to the wealthy information from the precision lesion localization in SGD-Net Plus. An advanced case analysis can efficiently bring the maximal professional skill gaining for single case studies. Integrating AI and clinical knowledge is a challenge for medical education (Chan and Zary, 2019), and a framework for stepwise education in professional development may shape the new generation to integrate and utilize AI-assisted information effectively (Paranjape et al., 2019). Domain knowledge is the way to unlock the full potential of AI tools (Masters, 2019). Indeed, practical orientation is the original intention behind the creation of SGD-Net Plus. When classical neurology collects symptoms and signs to localize lesions in the brain, SGD-Net Plus applies retrograde engineering by first knowing the lesion occupancy in the brain, degrading the affected regions and tracts, and then projecting to the patients' manifestations. Therefore, SGD-Net Plus strengthens and deepens the clinical-anatomical connections and generalizes brain MRI to general users. Its deployment will be helpful to professional educators, medical trainees, clinical physicians, and those who want to learn AIS lesion information from MRI images.

### 4.5 Limitations

There are several limitations to this study. First, although we have used chronologically separated datasets for validation, we did lack external validation datasets from different sites and different patient populations. Stroke patients in different geographic sites may present with varied stroke patterns because of demographic factors, genetic variance, and lifestyle. In addition, we did not test SGD-Net on datasets acquired from different MRI machines in the current study. The current dataset was collected from the same 3 T MRI machines and could not verify whether machine effects affect SGD-Net performance. We do not know if the model would be stable enough on images from 1.5 T MRI machines, which are still prevalent in clinical services. Therefore, multi-site validation is warranted to test the generalizability and stability of the SGD-Net.

Second, DWI had its resolution limitations to show small lesions, multiple scattered lesions, or low DWI intensity lesions. These imaging process-related restrictions would affect machine learning model performance. To reduce these confounding factors, we used images from 3 T MRI that produced higher quality in imaging production than 1.5 T MRI. Some other studies used integrative analysis of DWI and ADC images for AIS lesion segmentation. However, combining DWI and ADC did not improve AIS lesion segmentation much more than DWI alone (Winzeck et al., 2019). Therefore, we used 3 T MRI DWI alone as an acceptable modality for AIS lesion segmentation.

Third, we excluded patients with lesions in both anterior and posterior circulatory territories in location classification. Because of the low prevalence of patients with both territories in our dataset (N = 11/216, 5.1%, in Fig. 3), we excluded them from the current location classification study. However, a multi-class classifier is warranted for multiple-lesion localization for practical utility in the future.

Finally, we tested the SGD-Net and SGD-Net Plus only on retrospective datasets. Whether these tools improve real-world practice requires clinical deployment to evaluate their efficiencies in supporting clinical decision-making.

## 5 Conclusions

Combining automatic segmentation and classification of AIS is a developing field for applying deep learning in medical care. These applications are promising and may assist clinicians with interpreting brain MRI images in AIS patients and supporting clinical decision-making. SGD-Net for joint segmentation and classification of AIS lesions outperforms traditional 3D models for classifying AIS lesion size and location in DWI images. SGD-Net Plus, the modification of SGD-Net, realizes the precision localization of AIS lesions on standard brain atlases and provides informative reports to clinical physicians. Therefore, the auto-classifier can help those non-expert users to get information about AIS patients from MRI images. Future deployment of SGD-Net and SGD-Net Plus in medical care systems is promising to improve the quality of AIS patient care.

### CRediT authorship contribution statement

**Yi-Chia Wei:** Conceptualization, Validation, Investigation, Data curation, Writing – original draft, Visualization, Project administration, Funding acquisition. **Wen-Yi Huang:** Project administration, Funding acquisition. **Chih-Yu Jian:** Methodology, Software, Formal analysis, Data curation, Visualization. **Chih-Chin Heather Hsu:** Conceptualization, Software, Visualization, Writing – original draft. **Chih-Chung Hsu:** Methodology, Validation. **Ching-Po Lin:** Resources, Supervision. **Chi-Tung Cheng:** Methodology, Supervision. **Yao-Liang Chen:** Data curation. **Hung-Yu Wei:** Methodology, Supervision. **Kuan-Fu Chen:** Conceptualization, Methodology, Validation, Investigation, Writing – review & editing, Project administration, Funding acquisition.

## Declaration of Competing Interest

The authors declare that they have no known competing financial interests or personal relationships that could have appeared to influence the work reported in this paper.
